# Vitamin D and Diabetes in Pakistan: A Comprehensive Review of Existing Literature

**DOI:** 10.7759/cureus.106923

**Published:** 2026-04-12

**Authors:** Nayab Afzal, Amal Mahmood, Hafsa Majid, Lena Jafri, Aysha Habib H Khan

**Affiliations:** 1 Chemical Pathology, Aga Khan University Hospital, Karachi, PAK; 2 College of Medicine, Aga Khan University Hospital, Karachi, PAK; 3 Pathology and Laboratory Medicine/Chemical Pathology, Aga Khan University Hospital, Karachi, PAK; 4 Pathology and Laboratory Medicine, Aga Khan University Hospital, Karachi, PAK

**Keywords:** 25 (oh) vitamin d, pakistan, type 1 diabetes mellitus, type 2 diabetic mellitus (t2dm), vitamin-d deficiency, vitamin d levels (vit d level), vitamin d serum level, vitamin d status

## Abstract

Diabetes is rising rapidly across Pakistan, creating economic and managerial pressure on the healthcare system. At the same time, vitamin D deficiency is common throughout the country, creating a natural curiosity about how these two problems might intersect. Vitamin D is known to influence the body’s ability to manage glucose, yet studies from Pakistan have shown varied results. In this review, we included observational and interventional human studies conducted in Pakistan that measured serum vitamin D in relation to diabetes prevalence, glycemic control, or complications. A broad range of study designs, including cross-sectional, case-control, cohort, and randomized controlled trials, was considered to capture the diversity of available evidence. Most research came from urban centers and focused on adults with type 2 diabetes. Cross-sectional studies were the most common design, with fewer case-control, cohort, and interventional studies. A key issue across the literature was the lack of uniformity in how vitamin D levels were defined, with many studies using different thresholds or reporting styles. Genetic aspects of vitamin D deficiency were addressed in only a small number of studies. Despite these differences, most studies consistently found very low vitamin D levels among people living with diabetes in Pakistan. The findings point to a strong need for more standardized and methodologically robust research to better clarify how vitamin D fits into the broader picture of diabetes in the Pakistani population. A clearer, more cohesive evidence base will help guide better screening, treatment, and prevention strategies for people at risk.

## Introduction and background

Diabetes mellitus (DM) is recognized as one of the four leading non-communicable diseases characterized by high glucose levels due to insufficient insulin secretion or action. The International Diabetes Federation (IDF) estimated a 10.5% global prevalence of diabetes in adults aged 20-79 years in 2021 [1]. The prevalence is projected to increase further with more rapidly growing trends in lower and middle-income countries. It is estimated that between 2010 and 2030, there will be a 69% increase in adults living with DM compared to a 20% increase in developed countries [2]. Pakistan currently has an estimated 34.5 million adults (20-79 years) living with DM, with an estimated prevalence of DM in Pakistan at 31.4%, with the majority of diabetic individuals having poor glycemic control due to low socioeconomic status and education levels [3]. DM poses a very large burden on the healthcare system as it leads to many complications throughout the disease course.

Vitamin D has been implicated in many chronic diseases such as cancers, heart conditions, metabolic syndrome, autoimmune disorders, and even neurological diseases [4-6]. The relationship between vitamin D and DM has been established in several studies, with vitamin D supplementation delaying pancreatic β-cell decline and normalizing glycemic control, as evidenced by a reduction in HbA1c in diabetic patients [7]. The proposed reasoning behind this effect of vitamin D is its ability to influence insulin secretion and sensitivity through several mechanisms. It binds to receptors in pancreatic β cells and activates vitamin D-responsive elements in the insulin gene promoter. Intracellular calcium is crucial for insulin function, and vitamin D plays a key role in modulating calcium flux within pancreatic cells [8].

Vitamin D deficiency (VDD) is very common in South Asia, with Pakistan reporting the highest incidence [9]. There is a discrepancy in the literature between the relationship of vitamin D and DM. Ullmann et al. [10] reported that administering 50,000 IU of vitamin D weekly for eight weeks resulted in a significant decrease in proteinuria and creatinine levels in patients with diabetic kidney disease, with the most notable effects observed in individuals with type 2 DM. However, a meta-analysis found no significant reduction in HbA1c levels after vitamin D supplementation [11].

Preliminary observations from individual Pakistani studies also show inconsistent associations, underscoring the need for a comprehensive, critical synthesis of the national evidence base to inform local clinical and public health strategies. This comprehensive review synthesizes observational and interventional studies examining vitamin D and diabetes among the Pakistani population. The review aims to identify trends, highlight research gaps, guide future studies, and provide a comprehensive overview of the epidemiological and clinical associations between DM and vitamin D in the Pakistani population.

## Review

Methodology

A comprehensive literature review was conducted to identify and synthesize published evidence using Medline (PubMed interface) and PakMedinet databases on vitamin D and different types of DM. Relevant studies published between January 1984 and February 2025 were identified.

The search strategy used combinations of Medical Subject Headings (MeSH) terms and keywords related to vitamin D ((Vitamin D OR “Vitamin D deficiency” OR “25-hydroxyvitamin D”) AND (Diabetes OR “Type 2 diabetes” OR “Type 1 diabetes” OR “Diabetes mellitus” OR “Insulin resistance”), and geographic location (“Pakistan”). No restrictions were placed on language at the search stage. To ensure broad coverage of the literature, reference lists of relevant articles and the PubMed “related articles” feature were also reviewed.

A wide range of study designs was reviewed, including cross-sectional, case-control, observational, interventional, and longitudinal studies. Publications focusing on animal models, basic science research, or populations outside Pakistan were excluded. Case reports, conference abstracts without full manuscripts, editorials, and secondary literature such as reviews and meta-analyses were also excluded. Article selection and data extraction were undertaken to ensure accuracy and consistency, with disagreements resolved through discussion. The findings were synthesized narratively, with emphasis on identifying prevailing trends, methodological heterogeneity, and gaps in the existing evidence base.

Two researchers (AM and NA) reviewed the titles and abstracts independently before final analysis. Both reviewers worked independently and organized the data using Excel, enlisting the author(s) name (year)/city, type of study, sample size, type of study participants, vitamin D levels, method of analysis, and findings reported in the studies.

Results

From 1980 to 2024, a comprehensive search of relevant databases identified a broad range of studies examining vitamin D and diabetes in the Pakistani population. After applying the inclusion criteria, focusing on human studies conducted in Pakistan that reported vitamin D status in relation to diabetes, 38 studies were included in the review. The accumulated sample size of the studies comprised 8,957 individuals. The largest study had a sample size of 2,854 patients. Geographically, most studies were conducted in urban centers, with 16/38 (42.1%) from Sindh (primarily Karachi) and 14/38 (36.8%) from Punjab (mainly Lahore and Rawalpindi). Limited data were available from other provinces and rural areas.

The reviewed studies predominantly involved adults aged 18-65 years. Type 2 DM was the most studied, accounting for 32 (84.2%), while only a smaller proportion of studies examined type 1 DM or mixed diabetes populations. Cross-sectional studies were the most common design, representing 27 (71.1%) studies, followed by case-control studies at 6 (15.7%), while only a few were outcome-based. Only two randomized controlled trials (RCTs) and three quasi-experimental or open-label studies were identified, highlighting a major gap in high-quality interventional evidence. Various supplementation protocols of vitamin D administration were studied by 5 (13.2%) researchers. Of the 32 studies comparing diabetic and non-diabetic participants, 25 (78%) reported significantly lower mean or median serum 25(OH)D levels in individuals with diabetes. Interventional studies showed mixed effects of supplementation on glycemic control, while genetic studies were limited and inconclusive. Heterogeneity in vitamin D thresholds was substantial, with only 50% of studies following Endocrine Society Guidelines, as presented in Table 1.

**Table 1:** Summary of study characteristics and key findings on vitamin D Levels in diabetic populations in Pakistan. VD: vitamin D; VDR: vitamin D receptor

Feature	Number of studies	Key findings/Trend
Study design	Cross-sectional: 27; case-control: 6; cohort/interventional: 5	Cross-sectional studies dominated; interventional studies were few, but suggested VD supplementation may improve glycemic control
Studies reporting significantly lower VD in diabetics	25/32 (78%)	VD deficiency was common among diabetics
Adherence to standardized VD thresholds	19/38 (50%)	Endocrine Society Guidelines used; heterogeneity in definitions
Interventional studies (effect on HbA1c)	5	Three showed improvement; two showed no effect
Genetic studies (VDR polymorphisms)	4	Fok1 association was more consistent than Apa1/BsmI

Overall, these findings highlight the high prevalence of VDD among diabetics in Pakistan and underscore the need for standardized measurement and reporting in future research.

VDD was associated with poorer glycemic parameters, including higher fasting blood glucose, HbA1c, and impaired fasting glucose. In diabetic complications, low vitamin D levels were linked with a higher prevalence of neuropathy, retinopathy, and microalbuminuria. For example, 59% of patients with diabetic neuropathy had VDD versus 8% without neuropathy; 60% of type 2 DM patients with microalbuminuria had VDD compared to 40% with normo-albuminuria. Among five intervention studies, supplementation with vitamin D led to improvements in serum 25(OH)D, high-density lipoprotein cholesterol, calcium, and reductions in HbA1c in most studies. The effect on glycemic control varied depending on dose, baseline vitamin D levels, and patient characteristics. Three studies explored *VDR* gene polymorphisms. Associations with diabetes susceptibility were inconsistent; some studies reported significant links (e.g., *Fok1* polymorphism), while others found no correlation. We observed significant discrepancies in reporting of vitamin D levels as 19 (50%) adhered to the Endocrine Society Clinical Practice Guidelines, defining VDD as <20 ng/mL (or <50 nmol/L), vitamin D insufficiency (VDI) as 20-30 ng/mL (50-75 nmol/L), vitamin D sufficiency (VDS) or normal as >30 ng/mL (>75 nmol/L); 7(18%) did not specify thresholds. One study used stricter study-specific criteria (severe VDD: <10 ng/mL, moderate VDD: 5-20 ng/mL). One researcher used an unusual threshold of <8.2 ng/mL for VDD. When considering the reporting unit, ng/mL was used by an overwhelming number of studies, with only two studies reporting in nmol/L. Genetic aspects of VDD were studied by only three researchers. The majority of the studies revealed that there is a high prevalence of VDD among the diabetic population. The consistently high prevalence of VDD among Pakistani diabetic patients highlights the importance of regular screening and supplementation, particularly for high-risk individuals, as presented in Table 2.

**Table 2:** Summary of included studies in the systematic review of vitamin D and diabetes in Pakistan from 2012 to 2024. VD: vitamin D; VDS: vitamin D sufficiency; VDD: vitamin D deficiency; VDI: vitamin D insufficiency; VDT: vitamin D toxicity; DM: diabetes mellitus; T2DM: type 2 diabetes mellitus; T1DM: type 1 diabetes mellitus; IFG: impaired fasting glucose; FPG: fasting plasma glucose; 25(OH)D: 25-hydroxy vitamin D; HbA1c: hemoglobin A1c; RBS: random blood sugar; FBS: fasting blood sugar; OR: odds ratio; CI: confidence interval; SD: standard deviation; ANOVA: analysis of variance; QoL: quality of life; DN4: Douleur Neuropathique 4 (Neuropathic Pain Questionnaire); NeuroQoL: neuropathy quality of life; LDL: low-density lipoprotein; HDL: high-density lipoprotein; TG: triglycerides; ACR: albumin-to-creatinine ratio; VDR: vitamin D receptor; PTH: parathyroid hormone; PDN: painful diabetic neuropathy; DPN: diabetic peripheral neuropathy; NPDR: non-proliferative diabetic retinopathy; PDR: proliferative diabetic retinopathy; HL: hearing loss, ProDR: proliferative diabetic retinopathy; VDBP: vitamin D-binding protein; UACR: urine albumin-to-creatinine ratio

Author name (year)/City	Type of study, n	Study population	Serum 25(OH)D	Statistical analysis conducted or data analysis method	Key findings
Ijaz A et al. (2012) [12]/Rawalpindi	Case control, 86	Adults aged 19–65 years with T2DM	VDD: <20 ng/mL	Correlation between FPG and 25(OH)D levels was assessed using Pearson’s correlation coefficient	DM was more prevalent among individuals with VDD (50%) and VDI (31.6%) compared to those with normal VD levels (6.1%). Similarly, IFG was more common in VDD (25%) and VDI (26.3%) groups than in participants with normal VD status (18.6%). The odds ratio for VD in relation to DM was 3.36
Tariq et al. (2016) [13]/Rahimyar Khan	Cross-sectional, 190	97: newly diagnosed DM patients; 93: non-DM controls	VDD: <20 ng/mL	Correlation studies were carried out between FPG and 25(OH)D levels using Pearson’s correlation coefficient	A higher proportion of patients with DM had VDD (87%) and VDI (67%) groups compared to those with normoglycemia (13% and 33%, respectively)
Bashir et al. (2016) [14]/Karachi	Cross-sectional, 168	168 adults with T2DM	VDD: <20 ng/mL; VDI: 20–30 ng/mL; normal: >30 ng/mL	The relationship between glycemic control and vitamin D was computed through the chi-square test	Most patients were deficient in vitamin D3 (80.8%), 13.8% were insufficient, while 5.4% had sufficient vitamin D levels. VDD in FBS: >120 mg/dL, 85.2%; VDD in RBS: >140 mg/dL, 83.2%, HbA1c >7%; VDD: 86.5%, p-value: 0.001, 0.003, and 0.042
Nasreen et al. (2016) [15]/Lahore	Case control, 88	44 T1DM, 44 healthy controls	VDD: <10 ng/mL; VDI: 10–30 ng/mL; VDS: 30–150 ng/mL	Genotype frequencies were evaluated for Hardy-Weinberg equilibrium using the chi-square test. Quantitative variables were represented as mean and SD. The aAssociation between Fok1 and Apa1 and VD levels was assessed using Pearson’s chi-square and Fisher’s exact test, as appropriate	No statistically significant difference in VD levels between cases and controls. VDR polymorphism in the Fok1 and Apa1 genes was not associated with susceptibility to T1DM
Alam et al. (2017) [16]/Karachi	Prospective, 143	143 Patients with symptomatic diabetic neuropathy >18 years and HbA1c ≤11% with no comorbidities	VDD: <20 ng/mL; VDI: 20–30 ng/mL; VD adequacy: >30 ng/mL	Chi-square test or Student’s t-test was used for the analysis of anthropometric and metabolic parameters, and patients’ perception and general classification of QoL. The non-parametric Kruskal-Wallis test was utilized with post hoc analyses	Treatment with VD resulted in a significant increase in 25(OH)D, calcium, and HDL and a reduction in HbA1c (p = <0.05). Administration of a single high-dose treatment with vitamin D showed a significant improvement in the specific NeuroQol subscale of emotional distress, along with improvements in the self-classification of QoL
Bahir et al. (2017) [17]/Karachi	Cross-sectional, 168	168 adults with T2DM	VDD: <20 ng/mL; VDI: 21–30 ng/mL; normal: >30 ng/mL	Chi-square test for the relationship of dyslipidemia with glycemic control and dyslipidemia with VD status	VDD in cholesterol: >200 mg/dL 62.2%; VDD in LDL: >130 mg/dL 74.8%, VDD in HDL: <40 mg/dL 63.6%. P-value for LDL: 0.019; p-value for TG: 0.005
Khan et al. (2018) [18]/Peshawar	Cross-sectional, 100	50 DM and 50 controls	VDD: <8.2 ng/mL; normal to high: >8.2–37.4 ng/mL	Odds ratio for comparing cases and controls, confounding was evaluated using bivariate analysis, and values were determined using the chi-square or Fisher’s exact tests	VDD in cases: 18 (94%); VDD in controls: 1 (5%). Odds ratio = 18
Muneer et al. (2018) [19]/Lahore	Cross-sectional, 200	200 adults (12–30 years old) T1DM	VDD:<50 nmol/L; normal: >50 nmol/L	_	86.5% of T1DM patients had VDD; 13.5% had sufficient vitamin D levels
Iqbal et al. (2018) [20]/Karachi	Cross-sectional, 312	312 adults (22–70 years old) with T2DM	_	Multiple linear regression was used to observe the relationship between vitamin D levels and statin use	Statin use did not show any effect on circulating serum levels of vitamin D (p = 0.768)
Shafique et al. (2019) [21]/Karachi	Cross-sectional, 70	70 adults aged 30–75 years with T2DM and peripheral neuropathy	VDD: <20 ng/mL	_	VDD in 58.5% of peripheral neuropathy cases
Fatima et al. (2019) [22]/Faisalabad	Cross-sectional, 250	150 T2DM and 100 non-diabetics	_	All data were expressed as mean ± SD % or n (number)	No significant association was found between the VDR gene BsmI polymorphism and T2DM in a Pakistani population
Mehmood et al. (2019) [23]/Lahore	Case control, 100	75 patients in the age range of 40–50 years were divided into three groups with 25 patients in each group	_	The differences in the VDBP/Creatinine ratio among the groups was assessed using the independent Kruskal-Wallis test, followed by pairwise comparison was done with Dunn-Bonferoni test	The highest VDBP levels were in group 3 (diabetic patients with microalbuminuria), followed by group 2 (diabetic patients without microalbuminuria). Urinary ACR was highest in group 3
Kumar et al. (2019) [24]/Jamshoro	Observational study, 140	140 (35–60 years old); T2DM VDD: <30 ng/mL	VDD:<30 ng/mL	Student’s t-test was used	VD supplementation did not significantly impact HbA1c levels over 12 weeks
Hussain Gilani et al. (2019) [25]/Abbotabad	Cross-sectional, 109	58 patients with T2DM and 51 non-DM patients	VDS: >30 ng/mL; VDI: 20–30 ng/mL; VDD: <20 ng/mL	Significance testing in the case of categorical variables was done using the chi-square test	VD status in diabetic versus non-diabetic patients (p = 0.015). VDD in obese: 87.5%; VDD in non-obese: 51.2%; VDD in overweight: 56.5%; p-value = 0.018
Farooq et al. (2020) [26]/Karachi	Cross-sectional, 181	181 adults aged 40–70 years with T2DM for 3 years	VDD: <20 ng/mL; VDI: 20.1–29.9 ng/mL; normal->30 ng/mL	Chi-square test or Fisher’s exact test for the association between vitamin D, DR, and hearing status was applied	VDI: 14 (38.9%); mon ProDR VDI in 2 (5.6%) ProDR VDD in 33 (32.7%) NPDR VDD in 15 (14.9%) PDR patients, VDI in 12 (33.3%), mild HL in 1 (2.8%) with moderate and severe HL. VDD in 85 (84.2%) with mild HL, 4 (4.0%) with moderate and severe HL patients. VD levels declined significantly as the severity of DR and HL, with a p-value <0.0001
Rana et al. (2020) [27]/Lahore	Cross-sectional, 115	115 patients aged 18–76 years suffering from DM or HTN	VDD: <20 ng/mL; VDI: 20–30 ng/mL	_	VDD in 97.3% patients
Nasreen et al. (2020) [28]/Karachi	Case-control study, 88	44 in the T2DM and 44 in the control group	VDS: 30–150 ng/mL; VDI: 10–30 ng/mL; VDD: <10 ng/mL	_	T2DM was found to be significantly associated with Fok1 polymorphisms of the VDR gene
Khan et al. (2020) [29]/Peshawar	Cross-sectional, 238	238 T2DM	_	_	VDD was inversely associated with T2DM
Farooq et al. (2021) [30]/Karachi	Retrospective study, 497	497 T2DM patients aged 41–70 years	Normal: >30 ng/mL; VDI: 20.1–29.9 ng/mL; VDD: <20 ng/mL	Chi-square test or Fisher’s exact test for the significance between vitamin D levels and the status of diabetic retinopathy	Normal VD: 17 (27.0%); NPDR VDI: 133 (53.2%); VDD: 115 (62.5%); VDI: 53.2% were having NPDR VDI 24.4% were having PDR
Memon et al. (2022) [31]/Karachi	Cross-sectional, 200	200 patients (25–65 years old) divided into two groups, T2DM patients (100) and non-diabetic controls (100)	_	Chi-square test of association was performed	Significant relationship between the Fok1 gene polymorphism of VDR and susceptibility to T2DM was found
Bashir et al. (2022) [32]/Karachi	Case control, 302	302 (35–65 years old), 151 healthy controls, and 151 newly diagnosed T2DM	VDD: <25 nmol/L; VDI: 25–50 nmol/L	Pearson correlation and linear regression curve estimation between vitamin D levels and HbA1c	VDD in T2DM in 58 (38.4%), VDI in 61 (40.4%), normal in 32 (21.2%), VDD in controls 13 (8.6%), VDI in 53 (35%), normal in controls 85 (56%); p-value <0.05
Khudayar et al. (2022) [33]/Karachi	Cross-sectional, 1050	525 T2DM (30–60 years old) 525 Controls	VDS: >30 ng/mL VDD: <30 ng/mL	Chi-square test for the association between VDD and T2DM	VDD 54.1% in cases, VDD 25.9% in controls; p-value = 0.001
Riaz et al. (2023) [34]/Rawalpindi	Cross-sectional, 110	110 patients diagnosed with T2DM aged between 30 and 65 years	VDS: >30 ng/mL; VDI: 20–30 ng/mL; VDD: <20 ng/mL	Chi-square test for the association of VD status with gender, age group, and albuminuria status. Pearson correlation for the correlation between the VD level and UACR	Serum VDD was greater in T2DM patients with microalbuminuria (60.42%) compared to T2DM patients with normoalbuminuria (39.58%)
Ahsan et al. (2024) [35]/Karachi	Retrospective, 2,854	_	VDT: >150 ng/mL; VDD: <20 ng/mL; VDI: <30 ng/mL; normal: >30 ng/mL	Chi-square and Fisher’s exact tests were applied. Binary logistic regression for the association between VDD and different non-communicable diseases	T2DM was associated with higher odds of sufficient vitamin D levels (OR = 1.266; p = 0.388). T2DM might be associated with higher odds of sufficient vitamin D levels. 18.6% of patients: normal VD levels 24.9% of patients: VDI 20.0% of patients: VDD 16.3% of patients: severe VDD
Parveen et al. (2024) [36]/Lahore	Cross-sectional, 20	Twenty families diagnosed with T2DM	_	Delving into the vitamin D receptor variation and expression profiles in the context of type 2 diabetes among families	The VDR gene was expressed at higher levels in diabetic patients compared to controls (non-diabetic)
Gul et al. (2024) [37]/Peshawar	Comparative cross-sectional study, 100	50 patients with diabetes and 50 controls (non-diabetics)	VDD: <10 ng/mL	Chi-square test and independent sample t-test	VDD was found more in the DM group 15 (30%) than in the control group
Jamali et al. (2023)[38]/Nawabshah	Cross-sectional, 58	58 diabetics aged 40–60 years	VD normal: >29 ng/mL; VDI: <20–29 ng/mL; moderately deficient: 20–5.0 ng/mL; severely deficient: <5 ng/mL	_	48.30% of the patients with moderately deficient VD level significant negative correlation between VD level and HbA1c
Zehra et al. (2023) [39](2024)/Karachi	Cross-sectional, 80	80 type 2 diabetics aged 18–60 years	Normal: >30 ng/mL (75 nmol/L); VDI: 20–30 ng/mL (50–75 nmol/L); VD absence: <20 ng/mL (50 nmol/L)	Chi-square test	No meaningful change was found in HbA1c levels before and after therapy. VD plays no significant role in decreasing FBS and HbA1c
Mahmood et al. (2023) [40]/Peshawar	Cross-sectional, 219	219 T2DM patients	VD normal: >30 ng/mL; VDI :21–29 ng/mL; VDD: <20 ng/mL	Chi-square test	VD levels declined as HbA1c levels increased, with a p-value of 0.002
Yasin et al. (2024) [41]/Lahore	Cross-sectional, 50	50 diabetics	_	Diabetic patients’ vitamin D and calcium levels were compared using a correlation analysis	Significantly lower VD levels in diabetes patients compared to the controls
Khan et al. (2023) [42]/Quetta	Cross-sectional, 319	319 individuals	VDS: 75–250 nmol/L; VDI: 25–75 nmol/L; VDD: <25 nmol/L	The Kruskal-Wallis test was used to compare the levels of Vitamin D and PTH among the three groups	Prediabetic group, VDI: 27%, VDD: 81%. Diabetic group, VDI: 24%; VDD: 73%
Hidayat et al. (2023) [43]/Peshawar	Case-control study, 98	49 cases and 49 controls	VDD: <20 ng/mL; VDI: 20–30 ng/mL	Logistic regression analysis was used to see the association between quantitative attributes	59.2% patients with DNP had VDD compared to 8.2% diabetics with no DPN
Arain et al. (2024) [44]/Nawabshah	Cross-sectional study, 195	195 T2DM patients aged 18 to 70 years	_	Qualitative data were analyzed using the chi-square test, and quantitative variables were analyzed using the independent sample t-test	47.1% of patients with poor glycemic control had VDD compared to good glycemic control patients

Among the five interventional studies (two RCTs, two quasi-experimental studies, and one open-label study), improvements in neuropathic pain scores and quality-of-life measures were reported, indicating a potential role of vitamin D supplementation in diabetic neuropathy beyond glycemic control. However, findings related to glycemic outcomes were inconsistent. Some studies found no significant effect of supplementation on HbA1c, underscoring variability in metabolic responses. In contrast, other studies demonstrated significant reductions in HbA1c following vitamin D supplementation, particularly among participants with baseline VDD, suggesting a possible benefit in selected subgroups. Collectively, Pakistani interventional studies suggest a modest and context-dependent benefit of vitamin D supplementation, with more consistent effects observed for neuropathy-related outcomes than for glycemic indices such as HbA1c. Differences in baseline vitamin D status, dosing regimens, duration of supplementation, and outcome assessment likely contribute to the variability in results, as presented in Table 3.

**Table 3:** Description of randomized controlled trials, quasi-experimental, and open-label studies on vitamin D and diabetes in Pakistan from 2012 to 2024. T2DM: type 2 diabetes mellitus; VD: vitamin D; VDS: vitamin D sufficiency; VDD: vitamin D deficiency; VDI: vitamin D insufficiency; DN4: Douleur Neuropathique 4 (Neuropathic Pain Questionnaire); HbA1c: hemoglobin A1c

Author name (year)/City	Type of study/Sample size, n	Study population	Vitamin D levels	Method of data analysis	Key findings
Basit et al. (2016) [45]/Karachi	Open-label study, 143	143 participants with predominantly T2DM	VDD: <20 ng/mL; VDI: 20–30 ng/mL; VDS >30 ng/mL	ANOVA and paired Student’s t-test or non-parametric counterpart were used depending on the normality of the data. Regression analyses were undertaken between baseline 25(OH)D status and total McGill pain location, McGill pain score, DN4, and positive symptoms at baseline	The group with <30 ng/mL showed significant improvements in more measures compared to the group with >30 ng/mL. The administration of 600,000 IU of vitamin D resulted in a modest but significant rise in 25(OH)D levels measured at 20 weeks
Randhawa et al. (2017) [46]/Lahore	RCT, 114	114 adults (35–50) with T2DM	_	ANOVA/Friedman test for: HbA1C level in both groups. Paired sample t-test/Wilcoxon signed-ranked test to compare the HbA1C level before and after treatment. Chi-square test: efficacy of treatment was assessed by using	No clinically significant impact on HbA1c reduction in T2DM
Akram et al. (2023) [47]/Faisalabad	Quasi-experimental, 30	30 T2DM with VDD	VDI: 21–30 ng/mL; VDD: <20 ng/mL	The paired Student’s t-test was used to compare data before and after the intervention	VD supplementation in T2DM patients can improve the serum VD level and also decrease proteinuria
Fawad et al. (2024) [48]/Karachi	RCT, 159	159 diabetics with type 1 or type 2 diabetes, aged 25–80 years	VDS: >30 ng/mL; VDI: 21–29 ng/mL; VDD: <20 ng/mL	Chi‐square test and ANOVA were used. Pearson’s correlation assessed the relationship between vitamin D levels and other parameters	VD supplementation in patients with PDN increases vitamin D levels, DN4 score, and HbA1c; however, these changes were insignificant
Javaid et al. (2024) [49]/Rawalpindi	Quasi-experimental trial, 100	100 patients between the ages of 20 and 65 years	VDD: <20 ng/dL	Paired sample t-test was used to assess the mean differences in HbA1c levels before and after the vitamin D treatment	For patients with baseline VD levels ≤ 40 nmol/L, the mean HbA1c declined from 8.44 ± 1.78 at the beginning to 7.37 ± 1.09 after three months. Among those with baseline VD levels >40 nmol/L, mean changes in HbA1c were 8.64 ± 1.92 initially, 7.55 ± 1.04 three months follow-up

Discussion

Pakistan currently has the highest incidence of VDD in South Asia [50]. Many studies have implicated the relationship of vitamin D and DM due to the crucial role of vitamin D in the regulation of insulin secretion from the pancreatic β cells and modulation of glucose metabolism. Vitamin D promotes expression of the human insulin receptor gene, thereby influencing insulin function and insulin sensitivity [51].

In this systematic review, we found studies that demonstrated the role of vitamin D supplementation on different diabetes parameters, such as HbA1c and fasting glucose levels, or on post-diabetic complications, such as neuropathy, nephropathy, and retinopathy [16,24,39,45-49]. Javaid et al. reported a notable decrease in HbA1c levels after vitamin D supplementation [49]. Basit et al. reported that both groups experienced significant reductions in pain and neuropathic symptoms, with greater improvements observed in individuals with lower vitamin D levels (<30 ng/mL) after a single dose of vitamin D [45]. Alam et al. concluded that administration of a single high dose of vitamin D produces a significant reduction in HbA1c and Neuro4 score, thus improving diabetic neuropathy pain [16]. These results align with the findings of Karonova et al., who reported that vitamin D supplementation for 24 weeks resulted in improvement in neuropathic symptoms [52]. The study by Akram et al. reported that oral vitamin D 50,000 IU weekly for eight weeks decreased proteinuria in diabetic patients [47].

Genetic associations were explored in a limited number of studies (n = 4), primarily focusing on VDR polymorphisms. Across the included studies, the *Fok1* polymorphism emerged as the most consistently associated variant with diabetes susceptibility in the Pakistani population, particularly in type 2 DM. Results from genetics studies done by Nasreen et al., checking for VDR polymorphisms in *Fok1* and *Apa1* genes, revealed no association with susceptibility to type 1 DM [15]. Nasreen et al. published another case-control study evaluating the VDR gene polymorphism in type 2 DM, whereby a significant association was found between the two in a Pakistani population [28]. Another similar study on VDR gene polymorphism was done by Memon et al., who found a significant relationship between *Fok1* gene polymorphism of VDR and susceptibility to type 2 DM [31]. These findings are consistent with the studies done in the UAE and Morocco [53,54]. A meta-analysis by Lei et al. investigated the link between four VDR polymorphisms and type 2 DM, identifying a significant association between the *Fok1* homozygous ff genotype and increased T2D risk [55]. The study concluded that the *Fok1* VDR polymorphism could be a risk factor for type 2 DM, particularly notable in the Asian population. In contrast, studies by Selvarajan et al. and Hatmal et al. found no association of VDR gene variants (*Fok1*, *Taq1*, *Apa1*) with type 2 DM [56,57]. Fatma et al. reported VDR *BsmI* polymorphism was not associated with type 2 DM in the Pakistani population [22]. However, this finding is inconsistent with a meta-analysis study performed by Wang et al. that highlighted a link between the *BsmI* polymorphism in the VDR gene and an increased risk of type 2 DM [58]. Ghomlani et al. observed that the VDR *BsmI* polymorphism was linked to a higher risk of insulin resistance in children and adolescents. [59]. The current available literature shows many inconsistent findings regarding the association of VDR gene polymorphisms and the development of DM. These discrepancies may stem from genetic diversity among populations and a limited understanding of the underlying biological mechanisms. Taken together, the genetic evidence suggests a possible population-specific role of the VDR *Fok1* polymorphism in type 2 DM susceptibility, although the small number of studies and limited sample sizes preclude definitive conclusions. These findings align with regional and Asian meta-analyses reporting stronger *Fok1* associations compared to other VDR variants.

There were many cross-sectional studies (n = 22) conducted throughout Pakistan investigating the prevalence of vitamin D levels in diabetics. The collective findings suggest a high prevalence of VDD among individuals with diabetes, with most studies reporting a significant inverse association between vitamin D levels and glycemic control. However, some studies presented conflicting results, highlighting the complexity of this relationship. The majority of studies reviewed demonstrated that individuals with diabetes had significantly lower serum vitamin D levels compared to non-diabetic controls [13,14,17-19,21,25-27,29,33,37,41,42]. Several studies also reported an association between lower vitamin D levels and poor glycemic control, with HbA1c levels inversely correlated with vitamin D concentrations [14,38,40,44].

Additionally, only one study by Khan et al. explored the role of VDD in prediabetic individuals, despite emerging evidence that deficiency may contribute to early metabolic dysfunction [42,60]. The findings from Pakistan align with global trends, where VDD has been implicated in diabetes pathogenesis. Both diabetes and VDD share many modifiable risk factors in the Pakistani population, including limited sun exposure, inactive lifestyle, and dietary habits.

In addition to investigating the association between VDD and DM, a subset of studies explored its relationship with diabetes-related complications, including microalbuminuria, diabetic retinopathy, and other vascular complications. Several studies reported a dose-response relationship, with declining vitamin D levels paralleling worsening glycemic control. A cross-sectional study by Bashir et al [17]. looking at dyslipidemia and its association with serum vitamin D levels in diabetics found that high total cholesterol, low-density lipoprotein cholesterol, and triglycerides show a significant association with VDD. Shafique et al. highlighted that patients with type 2 DM who had VDD were at a higher risk of experiencing early onset of diabetic peripheral neuropathy [21]. Approximately 59% of patients with diabetic peripheral neuropathy had vitamin D deficiency compared to only 8% of diabetics without neuropathy. Another cross-sectional study by Farooq et al. reported that low serum levels of vitamin D were associated with increased severity of diabetic retinopathy and hearing loss in type 2 diabetics [26]. The significant association of a decline in serum vitamin D levels and an increased occurrence of microalbuminuria in individuals with type 2 DM was noted by Riaz et al. [34]. Overall, 60% of patients with diabetic microalbuminuria were vitamin D deficient versus 40% with normoalbuminuria. These findings are similar to the available literature showing that VDD leads to early development of diabetes related complications [61].

Our review identified a few limitations in the included studies. In this literature review, we noted an overwhelming majority of studies utilized a cross-sectional study design focusing on the prevalence of VDD in various pathological conditions. This limits causal inference, which is critically important for identifying whether VDD exacerbates diabetes or results from it. A lack of robust study designs (RCTs, case-control studies) was also noted, as shown in Table 3. This highlights a need for more interventional and longitudinal research to establish causal relationships between VDD and diabetes. The adult population group (18-60 years) was overrepresented, while more work is required in pediatric/geriatric populations. Heterogeneity of VD classification ranges led to the inability to conduct a robust pooled analysis. We recommend that future studies use better sampling techniques with a detailed explanation of the study methodology. Further research is needed focusing on the pediatric and geriatric populations to gain an understanding of the role of vitamin D in diabetes across the lifespan.

## Conclusions

Based on our systematic review of the available literature, we conclude that vitamin D plays a meaningful role in the pathogenesis of DM in the Pakistani population, with evidence linking deficiency to poor glycemic control and early diabetes-related complications. While vitamin D supplementation has shown benefits in neuropathy and nephropathy. Genetic evidence indicates that the *FokI* polymorphism of the *VDR* gene is associated with an increased risk of type 2 DM, whereas no such association has been observed for type 1 DM. Although it is to be noted that 38 studies included in this review had variations in their study designs. VDD is commonly noted in diabetics in Pakistan, suggesting a potential role in its pathogenesis. Further studies are needed to establish causality.

## References

[1] Sun H, Saeedi P, Karuranga S (2022). IDF Diabetes Atlas: global, regional and country-level diabetes prevalence estimates for 2021 and projections for 2045. Diabetes Res Clin Pract.

[2] Liu J, Bai R, Chai Z, Cooper ME, Zimmet PZ, Zhang L (2022). Low- and middle-income countries demonstrate rapid growth of type 2 diabetes: an analysis based on Global Burden of Disease 1990-2019 data. Diabetologia.

[3] International Diabetes Federation (2025). Diabetes Atlas.

[4] Argano C, Mirarchi L, Amodeo S, Orlando V, Torres A, Corrao S (2023). The role of vitamin D and its molecular bases in insulin resistance, diabetes, metabolic syndrome, and cardiovascular disease: state of the art. Int J Mol Sci.

[5] Peixoto RD, Oliveira LJ, Passarini TM (2022). Vitamin D and colorectal cancer - a practical review of the literature. Cancer Treat Res Commun.

[6] Piędel F, Rocka A, Piwek M, Jasielski PP, Petit V, Rejdak K (2021). Correlation between vitamin D and alterations in MRI among patients with multiple sclerosis. Ann Agric Environ Med.

[7] Cojic M, Kocic R, Klisic A, Kocic G (2021). The effects of vitamin D supplementation on metabolic and oxidative stress markers in patients with type 2 diabetes: a 6-month follow up randomized controlled study. Front Endocrinol (Lausanne).

[8] Zhao H, Zheng C, Zhang M, Chen S (2021). The relationship between vitamin D status and islet function in patients with type 2 diabetes mellitus. BMC Endocr Disord.

[9] Siddiqee MH, Bhattacharjee B, Siddiqi UR, Rahman MM (2022). High prevalence of vitamin D insufficiency among South Asian pregnant women: a systematic review and meta-analysis. Br J Nutr.

[10] de Oliveira E Silva Ullmann T, Ramalho BJ, Laurindo LF (2023). Effects of vitamin D supplementation in diabetic kidney disease: a systematic review. J Ren Nutr.

[11] Li X, Liu Y, Zheng Y, Wang P, Zhang Y (2018). The effect of vitamin D supplementation on glycemic control in type 2 diabetes patients: a systematic review and meta-analysis. Nutrients.

[12] Ijaz A, Yousaf S, Ali M (2012). Association of vitamin D status and diabetes mellitus. J Bahria Univ Med Dent Coll.

[13] Tariq S, Majeed Z, Ghafoor MT (2016). Association of vitamin D deficiency and new onset type-2 diabetes mellitus. Pak J Pathol.

[14] Bashir F, Khan ZU, Qureshi S, Seetlani NK, Sheikh Z (2016). Prevalence of hypovitaminosis D in type 2 diabetes mellitus and its relationship with glycemic control. J Liaquat Uni Med Health Sci.

[15] Nasreen M, Lone KP, Khaliq S, Khaliq S (2016). Serum vitamin D levels and gene polymorphisms (Fok1 and Apa1) in children with type I diabetes and healthy controls. J Pak Med Assoc.

[16] Alam U, Fawwad A, Shaheen F, Tahir B, Basit A, Malik RA (2017). Improvement in neuropathy specific quality of life in patients with diabetes after vitamin D supplementation. J Diabetes Res.

[17] Bashir F, Khan ZU, Seetlani NK, Sheikh Z (2017). Pattern of dyslipidaemia and its association with hypovitaminosis D in type 2 diabetes mellitus. J Ayub Med Coll Abbottabad.

[18] Khan F, Usman R, Wadud S, Zafar S, Abideen ZU (2015). Association of vitamin D with retinopathy in patients with type 2 diabetes mellitus. J Med Sci.

[19] Muneer K, Hashmat N, Ayub T, Ahad UA (2018). Diabetes mellitus: frequency of vitamin D deficiency in type I diabetes mellitus. Prof Med J.

[20] Iqbal K, Islam N, Azam I, Mehboobali N, Iqbal MP (2018). Lack of association of statin use with vitamin D levels in a hospital based population of type 2 diabetes mellitus patients. Pak J Med Sci.

[21] Shafique S, Ansari R, Ariff H, Jafri SA (2019). Association of vitamin D deficiency with type 2 diabetes. J Bahria Univ Med Dent Coll.

[22] Fatma H, Abdul SN (2019). Association of Vitamin D receptor gene BsmI polymorphism with type 2 diabetes mellitus in Pakistani population. Afr Health Sci.

[23] Mehmood HMK, Yasmeen M, Ghaznavi S, Waheed MA, Rasheed MN (2019). Comparison of urinary vitamin D binding protein with albumin-creatinine ratio in type 2 Diabetes Mellitus as an early screening tool for diabetic nephropathy. J Fatima Jinnah Med Univ.

[24] Kumar G, Laghari J, Memon S, Qazi N, Shahani MY, Jamali S (2019). Effect of vitamin D supplementation on glycosylated hemoglobin in patients of type 2 diabetes mellitus taking oral antidiabetic drug metformin. Prof Med J.

[25] Hussain Gilani SY, Bibi S, Siddiqui A, Ali Shah SR, Akram F, Rehman MU (2019). Obesity and diabetes as determinants of vitamin D deficiency. J Ayub Med Coll Abbottabad.

[26] Farooq MUZ, Inamullah Syed, Mashhood S, Rana MA, Fahim MF (2020). Deficiency of vitamin D: influence on diabetic retinopathy and hearing loss among patients with diabetes mellitus type 2. J Bahria Univ Med Dental Coll.

[27] Rana MA, Javed M, Zartash S, Chaudhary A, Alam H (2019). Prevalence of vitamin D deficiency in patients with chronic diseases like diabetes and hypertension presenting to Medicine Clinic in Bahria International Hospital, Lahore, Pakistan. Pak Postgrad Med J.

[28] Sattar NA, Hussain F (2020). Vitamin D receptor FokI polymorphism in a Pakistani population with type 2 diabetes mellitus. Pak J Pharm Sci.

[29] Khan Z, Khan M, Bahadur S, Khan Z, Khan Y, Umair H, Jan SS (2020). Vitamin-D level in patients with type-2 diabetes mellitus of different age and sex: its effects on BMI, calcium level. J Saidu Med Coll Swat.

[30] Farooq MZ, Inamullah S, Mashhood S (2022). Evaluation of correlation between diabetic retinopathy and hearing impairment in type 2 diabetes mellitus. Life Sci.

[31] Memon MA, Baig S, Siddiqui PQ (2022). Fok1 VDR gene polymorphisms as the risk factor for diabetes mellitus. J Coll Physicians Surg Pak.

[32] Bashir Z tul-A, Amjad H, Gilani STA, Khan A, Riaz SU (2020). Frequency of vitamin D deficiency in newly diagnosed type 2 diabetes mellitus patients. Nat J Health Sci.

[33] Khudayar M, Nadeem A, Lodi MN (2022). The association between deficiency of vitamin D and diabetes mellitus type 2 (DMT2). Cureus.

[34] Riaz MH, Jamil A, Yousaf H (2023). Incidence of vitamin D deficiency and its association with microalbuminuria in patients with type 2 diabetes mellitus. Cureus.

[35] Ahsan T, Ghaus S, Sohail E, Aijaz W, Erum U, Jaffri SA (2024). Vitamin D status of patients visiting a private endocrinology clinic: a retrospective analysis from Karachi, Pakistan. J Pak Med Assoc.

[36] Parveen A, Batool A, Wajid A (2024). Delving the vitamin D receptor variation and expression profiles in the context of type 2 diabetes among families. Mol Biol Rep.

[37] Gul A, Amir M, Arshad AR, Qayyum S, Khan AH, Sardar A (2024). Association of vitamin D deficiency and type-2 diabetes mellitus in adults. Pak Armed Forces Med J.

[38] Jamali AA, Memon NA, Rabbani U, Komal Komal, Soomro AK, Jahanghir S (2023). Vitamin D deficiency in the patients of diabetes mellitus and its correlation with HbA1c. Ann Punjab Med Coll.

[39] Zehra R, Naz Z, Mohyuddin S, Zainab S, Amjad A, Hussain I (2024). Effect of vitamin D on fasting blood sugar and HbA1c in type 2 diabetes mellitus patients. J Aziz Fatimah Med Dent Coll.

[40] Mahmood A, Khan NA, Ahmad S, Naz S, Ahmed F, Khan AJ (2023). Association of serum vitamin D with glycosylated hemoglobin levels and duration of disease in type-II diabetes mellitus patients. J Med Sci.

[41] Yasin T, Khan M, Iqbal H, Azam H, Sukhera S, Arif HA (2024). Correlation of vitamin D and calcium levels and their biochemical importance in diabetic patients. Esculapio J SIMS.

[42] Khan A, Asif N, Khan TA, Khan A, Younas M, Ain QU (2023). Comparison of parathyroid hormone and vitamin D levels in individuals with prediabetes, diabetes and normoglycemia. Pak Armed Forces Med J.

[43] Hidayat M, Jehandad K, Muhammad A, Abideen ZU, Sabir U, Haq M (2023). Association of vitamin D level with diabetic peripheral neuropathy. Liaquat Natl J Prim Care.

[44] Arain N, Khuhro BA, Chandio MA, Shaikh NS, Kazi S, Bhagwani Bhagwani, Kazi SA (2024). Relationship of vitamin D levels and glycemic control in patients with type 2 diabetes mellitus. J Pak Univ Med Health Sci.

[45] Basit A, Basit KA, Fawwad A (2016). Vitamin D for the treatment of painful diabetic neuropathy. BMJ Open Diabetes Res Care.

[46] Randhawa FA, Mustafa S, Khan DM, Hamid S (2017). Effect of Vitamin D supplementation on reduction in levels of HbA1 in patients recently diagnosed with type 2 diabetes mellitus having asymptomatic vitamin D deficiency. Pak J Med Sci.

[47] Akram A, Khan AM, Anwar O, Arif M, Anjum A (2023). The effect of vitamin D supplementation on proteinuria in type 2 diabetes mellitus patients in DHQ Hospital Faisalabad. Pak J Med Health Sci.

[48] Fawwad A, Basit KA, Zafar AB (2023). Effect of single-dose oral vitamin D (200,000 IU) for the treatment of painful diabetic neuropathy. J Diabetol.

[49] Javaid Y, Sikandar R, Wasif A (2024). Effect of vitamin D supplements in improving glycemic control in type 2 diabetes mellitus. J Rawalpindi Med Coll.

[50] Salim N, Abdul Sattar M, Adnan A (2022). High prevalence of vitamin D deficiency in Pakistan and miscarriages: a hazard to pregnancies. Ann Med Surg (Lond).

[51] Grammatiki M, Karras S, Kotsa K (2019). The role of vitamin D in the pathogenesis and treatment of diabetes mellitus: a narrative review. Hormones (Athens).

[52] Karonova T, Stepanova A, Bystrova A, Jude EB (2020). High-dose vitamin D supplementation improves microcirculation and reduces inflammation in diabetic neuropathy patients. Nutrients.

[53] Safar HA, Chehadeh SE, Abdel-Wareth L, Haq A, Jelinek HF, ElGhazali G, Anouti FA (2018). Vitamin D receptor gene polymorphisms among Emirati patients with type 2 diabetes mellitus. J Steroid Biochem Mol Biol.

[54] Errouagui A, Benrahma H, Charoute H, Barakat H, Kandil M, Rouba H (2014). Vitamin D receptor gene polymorphisms and vitamin D status and susceptibility to type 2 diabetes mellitus in Moroccan patients. Int J Sci Res Publ.

[55] Li L, Wu B, Liu JY, Yang LB (2013). Vitamin D receptor gene polymorphisms and type 2 diabetes: a meta-analysis. Arch Med Res.

[56] Selvarajan S, Srinivasan A, Surendran D, Mathaiyan J, Kamalanathan S (2021). Association of genetic polymorphisms in vitamin D receptor (ApaI, TaqI and FokI) with vitamin D and glycemic status in type 2 diabetes patients from Southern India. Drug Metab Pers Ther.

[57] Hatmal MM, Abderrahman SM, Nimer W (2020). Artificial neural networks model for predicting type 2 diabetes mellitus based on VDR gene FokI polymorphism, lipid profile and demographic data. Biology (Basel).

[58] Wang Q, Xi B, Reilly KH, Liu M, Fu M (2012). Quantitative assessment of the associations between four polymorphisms (FokI, ApaI, BsmI, TaqI) of vitamin D receptor gene and risk of diabetes mellitus. Mol Biol Rep.

[59] Gholami A, Montazeri-Najafabady N, Karimzadeh I, Dabbaghmanesh MH, Talei E (2024). The effect of BsmI (rs1544410) single nucleotide polymorphism of vitamin D receptor (VDR) on insulin resistance in healthy children and adolescents: a cross-sectional study. BMC Pediatr.

[60] Zarrin R, Ayremlou P, Ghassemi F (2024). The effect of vitamin D supplementation on the glycemic status and the percentage of body fat mass in adults with prediabetes: a randomized clinical trial. Iranian Red Crescent Med J.

[61] Zhou T, Shen L, Li Z (2022). Severe 25-hydroxyvitamin D deficiency may predict poor renal outcomes in patients with biopsy-proven diabetic nephropathy. Front Endocrinol (Lausanne).

